# Joint space width of the tibiotalar joint in the healthy foot

**DOI:** 10.1186/s13047-015-0086-5

**Published:** 2015-07-03

**Authors:** Kan Imai, Kazuya Ikoma, Masamitsu Kido, Masahiro Maki, Hiroyoshi Fujiwara, Yuji Arai, Ryo Oda, Daisaku Tokunaga, Nozomu Inoue, Toshikazu Kubo

**Affiliations:** Department of Orthopaedics, Graduate School of Medical Science, Kyoto Prefectural University of Medicine, Kawaramachi-Hirokoji, Kamigyo-ku, Kyoto 602-8566 Japan; Department of Orthopedic Surgery, Rush University Medical Center, Kawaramachi-Hirokoji, Kamigyo-ku, Kyoto 602-8566 Japan

**Keywords:** Ankle joint, Computed tomography, X-ray

## Abstract

**Background:**

It is important to evaluate dynamic changes in the joint space width of the ankle mortise in detail in order to better understand the pathology of foot and ankle disorders. However, there are few reports on changes in the joint space width of the foot and ankle assessed using 3D images. The purpose of this study was to determine the changes in the joint space width of the ankle (tibiotalar joint) in association with dorsiflexion and plantar flexion of the ankle joint in healthy feet.

**Methods:**

Computed tomography (CT) images of 10 healthy feet were obtained in the neutral, plantarflexed and dorsiflexed positions of the ankle joint, from which 3D virtual models were fabricated of the tibia, fibula and talus. The 3D joint space width in these models was calculated using a custom made software program.

**Results:**

The joint space width increased in the order of dorsiflexion, neutral position and plantar flexion. Regarding the amount of change in dorsiflexion and plantar flexion relative to the neutral position, there were no significant differences in the middle-middle position. On the other hand, there were highly significant differences in the medial-anterior, medial-middle and medial-posterior positions.

**Conclusions:**

The joint space width of the ankle joint can be calculated accurately using 3D reconstruction images. Our findings should assist in clarifying pathology associated with movement of the ankle during the gait cycle based on changes in the joint space width in feet exhibiting disorders.

## Background

Features of osteoarthritis of the ankle (tibiotalar) joint include pain and loss of normal locomotion. Osteoarthritis of the ankle is commonly seen secondary to trauma, such as fractures and/or ligament injuries, inflammation and disorders following surgery (e.g., subtalar joint and triple arthrodesis) [1, 2]. In particular, in cases in which lateral instability of the ankle remains due to inadequate treatment of anterior talofibular ligament injury, where the talus moving back and forth during the gait cycle, stress concentrates on the articular surface of the medial side of the tibial plafond and medial malleolus, with subsequent progression of osteoarthritis of the ankle of the medial type [3]. In contrast, patients with pes planus, the everted hindfoot experiences loading stress concentrated on the lateral side of the ankle, and as a result, osteoarthritis of the ankle occurs more laterally [4].

Recently, in order to elucidate the pathology and aggravation of foot disorders, a study of contact pressure and contact area was conducted on loading of the ankle joint using cadavers [5]. In addition, other studies have measured the joint space width of the ankle joint using healthy subjects using two-dimensional (2D) analysis techniques [6–9]. However, it is difficult to precisely measure the joint space width using 2D methods, because the articular surface of the tibial plafond and trochlea of the talus are complex three-dimensional (3D) structures.

Due to recent advances in diagnostic imaging technology, it is now possible to measure the *in vivo* kinematics and/or joint space width using 3D models reconstructed from 2D computer tomography (CT) images [10–13]. However, there are few reports assessing the joint space width of the foot and ankle using 3D images. Kido *et al.* compared the joint space width of the ankle joint in healthy participants and participants with pes planus in a neutral position of the ankle and found a difference in the distribution of the minimal joint space between the flat and healthy feet [14]. At present, there are no reports regarding the joint space of the ankle joint in plantar flexion or dorsiflexion.

We hypothesised that the relative bone position between the tibia and talus differs in healthy feet compared to those with foot or ankle disorders, such as pes planus or anterior tibiotalar ligament injuries. We also hypothesised that the position of the tibia and talus changes based on whether the ankle is dorsiflexed or plantarflexed. This change increases stress on the tibiotalar joint, leading to progression of osteoarthritis of the ankle.

The purpose of this study was to clarify changes in the joint space width of the ankle joint in healthy feet in association with dorsiflexion and plantar flexion of the ankle by evaluating the differences between healthy feet and feet with disorders of the joint space width. In addition, we also wanted to assess the effects of changes in the alignment of the ankle joint on the change in the joint space width of the ankle in feet with disorders.

## Methods

This study was approved by the Medical Ethics Committee of Kyoto prefectural university of medicine (IRB No. ERB-C-81). All participants included in the study provided their informed consent. Ten healthy volunteers with no history of foot or ankle injuries and no instability of the foot or ankle were recruited (median age, 28.2 years; range, 21 to 35; 6 males and 4 females).

A custom-made ankle rotational device (Rakuhoku Prosthetic and Orthotic Manufacturing Co., Ltd, Kyoto, Japan), which did not lock the talocalcaneal joint, was used so that the CT scans could be obtained under the same conditions, with the foot held in maximal plantar flexion or dorsiflexion of the ankle joint. The foot plate was free floating, and the participant was instructed to maintain a maximally plantarflexed or dorsiflexed position using a custom-made ankle rotational device (Rakuhoku Prosthetic and Orthotic Manufacturing Co., Ltd, Kyoto, Japan) [10]. While it is easy to place a load in the neutral position, it is very difficult to place a load in maximal dorsiflexion or plantar flexion with high reproducibility. Therefore, we used ankle CT images obtained under non-loaded conditions in this study. CT scans of the foot and ankle were obtained with participants lying in the supine position and the device set on the foot. The positions of the foot and ankle were neutral in all axes, the line connecting the centre of the heel and the second metatarsal was vertical and the tibial shaft through the ankle centre was horizontal and parallel to the table of the CT scanner [15].

During the CT scanning, the knee and hip joints were in flexed positions (approximately 10 °) in order to maintain the foot in a non-loaded position. The CT images were acquired in the axial plane from 5 cm proximal to the tibiotalar joint to the plantar surface in 0.9 mm contiguous slices (140 kV, AEC, 5–10 s, 30 cm field of view, 512 × 512 matrix).

**Fig. 1:**
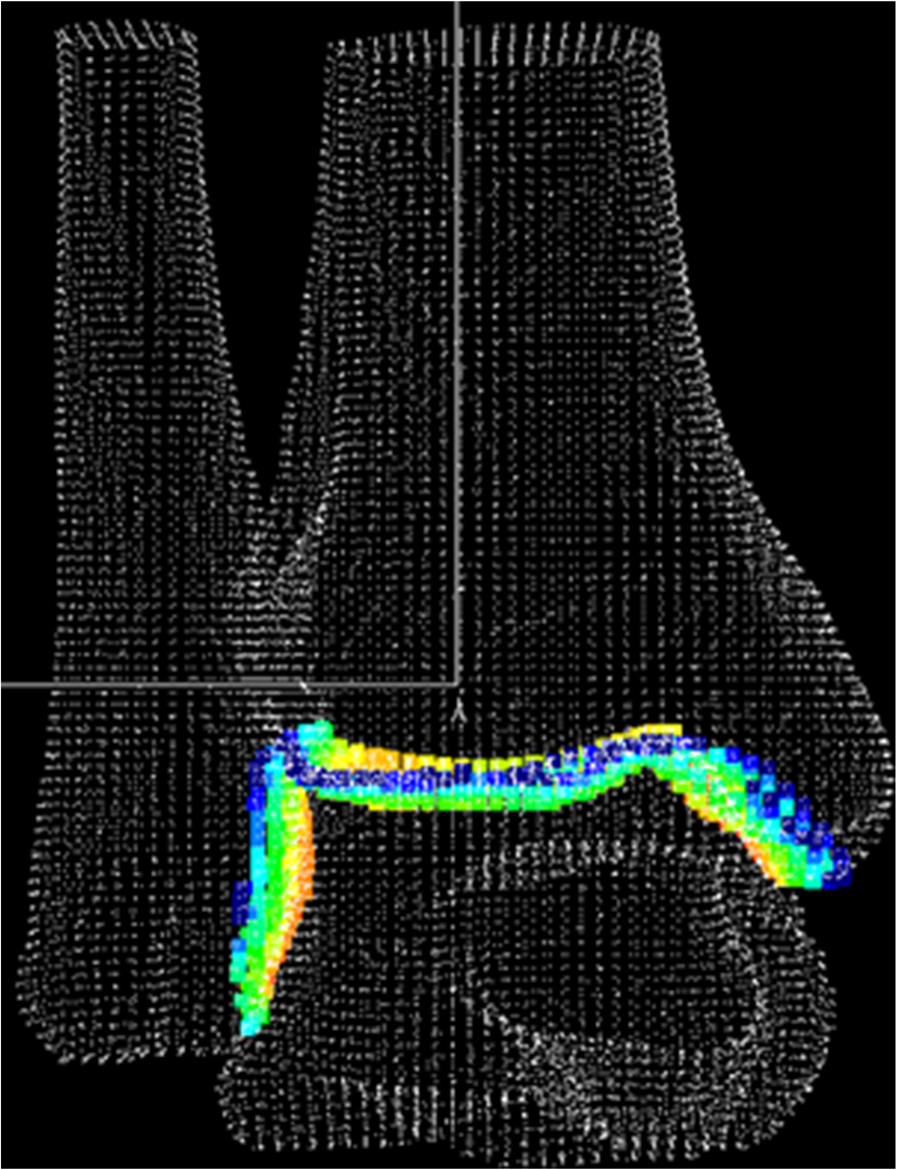
The procedures of joint surface tracing, triangulation, 3D reconstruction and area measurement were completed using a custom made software program

Subsequently, 0.9 mm-thick slices of axial CT scans obtained in the Digital Imaging and Communication in Medicine (DICOM) format were imported into a 3D reconstruction software package (Mimics; Materialise, Ann Arbor, MI, USA). The threshold level was selected to define the cortical shell, and the tibia, talus and fibula were segmented based on the threshold level. According to the most anterior and posterior coordinates of the tibial plafond, point-cloud data were detected and each tibial plafond point was divided into an anterior, middle and posterior one-third based on the anterior-posterior coordinate of each point. Similarly, the most medial and lateral coordinates of the tibial plafond point-cloud data were detected and each tibial plafond point was divided into medial, middle and lateral one-third based on the medio-lateral coordinate of each point. Consequently, the tibial plafond was equally divided into nine zones. Nine area measurement were completed using a custom made software program (Microsoft Visual C++ 2003 under Microsoft Foundation Class programming environment) [14] (Fig. 1).

**Fig. 2:**
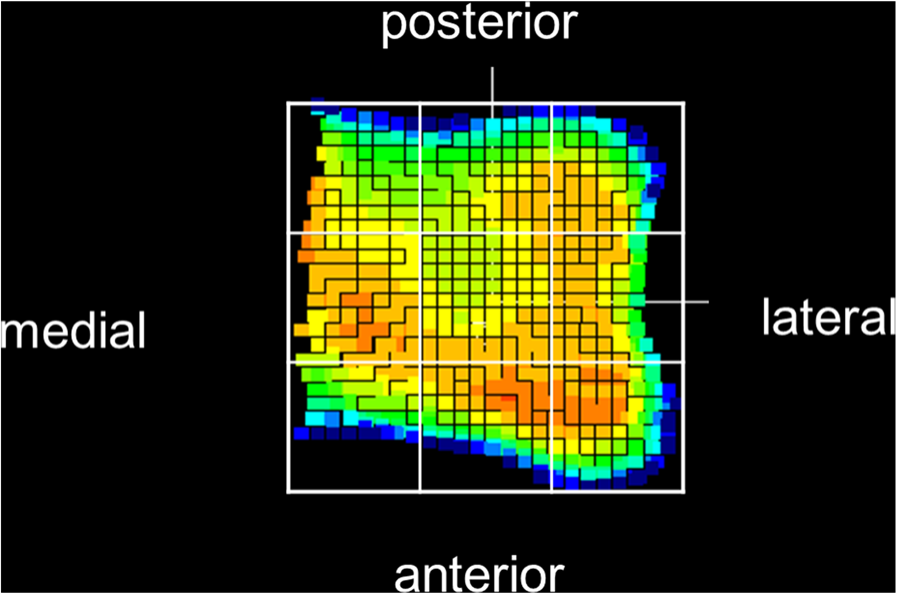
The tibial plafond was divided into nine areas (anterior-medial, anterior-middle, anterior-posterior, middle-medial, middle-middle, middle-lateral, posterior-medial, posterior-middle and posterior-lateral) based on coordinates of each point-cloud in the anterior-medial and medial-lateral direction

The mean values of the medial malleolus, tibial plafond and lateral malleolus were calculated in the neutral, plantar flexed and dorsiflexed positions. The tibial plafond was divided into nine areas (anterior-medial, anterior-middle, anterior-lateral, middle-medial, middle-middle, middle-lateral, posterior-medial, posterior-middle and posterior-lateral), and the mean joint space width for each area was calculated in three positions. Furthermore, the amount of change (i.e., the ratio) in the joint space width in plantar flexion and dorsiflexion was determined relative to that observed in the neutral position (Fig. 2).

The data are expressed as the mean ± standard deviation (SD). The paired *t*-test was used to compare the mean differences in the changes in the joint space width between dorsiflexion and plantar flexion. All analyses were performed using the StatView statistical software package (Version 5.0, SPSS, Chicago, IL, USA).

## Results

The joint space width in the neutral position for the medial malleolus was 1.70 ± 0.13 mm, while that for the tibial plafond was 2.04 ± 0.29 mm and that for the lateral malleolus was 2.13 ± 0.20 mm (Table 1). The joint space width increased in the order of dorsiflexion, neutral position and plantar flexion. Regarding the amount of change in dorsiflexion and plantarflexion relative to the neutral position, there were significant differences in each area (Table 2).

**Table 1:** Joint space width in mm (mean ± SD) of the tibiotalar joint

	Dorsiflexion	Neutral	Plantarflexion
Tibial plafond	1.95 ± 0.23	2.04 ± 0.29	2.37 ± 0.39
Medial malleolus	1.66 ± 0.11	1.70 ± 0.13	2.60 ± 0.50
Lateral malleolus	2.01 ± 0.19	2.13 ± 0.20	2.32 ± 0.29

**Table 2:** Comparisons of the ratio of tibiotalar joint space width (mean ± SD) between dorsiflexion and plantarflexion relative to the neutral position

	Dorsiflexion	Plantarflexion
Tibial plafond*	0.96 ± 0.07	1.17 ± 0.10
Medial malleolus*	0.98 ± 0.71	1.53 ± 0.28
Lateral malleolus*	0.95 ± 0.56	1.09 ± 0.12

Units of measurement are mm/mm (i.e., dorsiflexion/neutral and plantarflexion/neutral)

*Indicates a statistically significant difference (*p* < 0.05) between dorsiflexion and plantarflexion values

Among the nine tibial plafond areas, the joint space width was narrower in the middle part than in the medial or lateral part. Similarly, the joint space width was narrower in the middle part than in the anterior or posterior part (Tables 3 and 4). With respect to the amount of change in dorsiflexion or plantar flexion relative to the neutral position, there were no significant differences in the middle-middle position. In contrast, there were highly significant differences in the medial-anterior, medial-middle and medial-posterior positions.

**Table 3:** Joint space width in mm (mean ± SD) of nine tibial plafond areas

		Lateral	Middle	Medial
Posterior	Df	2.07 ± 0.99	1.99 ± 0.73	2.47 ± 0.57
	N	2.09 ± 0.98	1.93 ± 0.94	2.88 ± 0.84
	Pf	2.54 ± 0.60	2.57 ± 0.74	3.67 ± 0.64
Middle	Df	1.83 ± 0.38	1.72 ± 0.39	2.09 ± 0.38
	N	1.86 ± 0.30	1.83 ± 0.38	2.41 ± 0.34
	Pf	2.05 ± 0.58	1.73 ± 0.57	2.92 ± 0.73
Anterior	Df	2.08 ± 0.74	1.94 ± 0.26	2.08 ± 0.32
	N	2.14 ± 0.52	2.10 ± 0.44	2.28 ± 0.96
	Pf	2.72 ± 0.62	2.39 ± 0.47	3.67 ± 0.89

*Df* dorsiflexion, *N* neutral, *Pf* plantarflexion

**Table 4:** Comparisons of the ratio of tibiotalar joint space width (mean ± SD) between dorsiflexion and plantarflexion relative to the neutral position in nine tibial plafond areas

		Lateral	Middle	Medial
Posterior	Df	1.06±0.51*	1.18±0.59^NS^	0.89±0.22*
	Pf	1.32±0.38*	1.53±0.95^NS^	1.40±0.41*
Middle	Df	0.99±0.17^NS^	0.95±0.18^NS^	0.87±0.98*
	Pf	1.11±0.30^NS^	0.93±0.23^NS^	1.20±0.21*
Anterior	Df	0.95±0.25*	0.94±0.13*	1.04±0.26*
	Pf	1.40±0.58*	1.17±0.24*	1.86±0.70*

Units of measurement are mm/mm (i.e., dorsiflexion/neutral and plantarflexion/neutral)

*Df* dorsiflexion, *N* neutral, *Pf* plantarflexion

*Indicates a statistically significant difference (*p* < 0.05)

^NS^indicates a non-significant difference

## Discussion

It is difficult to precisely measure the joint space width of the ankle using 2D methods, because the articular surface of the tibial plafond and trochlea of the talus are complex 3D structures. DeAngelis reported the joint space width of the ankle joint to be 3.6 ± 0.6 mm on x-ray images [6]. Goker *et al.* also documented a medial joint space width of the tibial plafond of 2.56 ± 0.50 mm, compared to 2.45 ± 0.55 mm for the lateral plafond [7]. In the current study, the mean joint space width in the neutral position for the medial malleolus was 1.70 ± 0.13 mm, for the tibial plafond it was 2.04 ± 0.29 mm, and for the lateral malleolus it was 2.13 ± 0.20 mm. We measured the joint space using a custom made software program, with a reported error of measurement of 2 % or less [14]. We speculate that the joint space width was narrower in our study than in the previous study using x-ray images due to differences in physique based on population groups and variation in the measurement techniques [6, 7, 14].

Jonsson *et al.* measured the medial, middle and lateral parts of the joint space width of the tibial plafond in the anterior-posterior view and the anterior, middle and posterior parts of the joint space width in the lateral view using x-ray images [8]. The authors reported that the joint space width in the anterior-posterior view became narrower in the order of the medial, middle and lateral regions, and in the lateral view in the order of the middle, posterior and anterior regions [8]. However, in our study, the joint space width in the middle part was narrow in both views. We speculate that the difference between our study and that of Jonsson *et al.* is due to the fact that the tibial plafond and trochlea of the talus are complicated shapes and it is difficult to evaluate them precisely using x-ray.

Few reports have evaluated the joint space width in plantar flexion or dorsiflexion of the ankle. Farsø *et al.* measured the joint space width of the ankle joint in the neutral position and plantar flexion and reported the joint space width to be 3.0 ± 0.4 mm in the neutral position compared to 3.8 ± 0.5 mm in plantar flexion; that is, the joint space was wider in plantar flexion than in the neutral position [9]. We obtained the same result in our study. In dorsiflexion of the ankle joint, the anterior part of the trochlea of the talus telescopes into the ankle mortise. In contrast, in plantar flexion of the ankle joint, the posterior region of the trochlea of the talus, which is narrower than the anterior region, is a similar width to the ankle mortise. This result indicates that the change in joint stability has an impact on the change in the joint space width.

In our study, the joint space width in the middle-middle part was not significantly different in any of the positions of the ankle joint. This finding indicates that the ankle moves in the middle-middle position as a centre of rotation for the tibiotalar joint in patients with healthy feet. Kido *et al.* compared the joint space width in healthy feet with that observed in feet with pes planus in the neutral position and reported that the joint space width was narrower in the anterior-lateral, anterior-middle, middle-middle and middle-medial positions in the feet with pes planus than in the healthy feet [12]. This suggests that the loading area shifts more anteriorly and laterally in feet with pes planus than in healthy feet. Our findings should assist in clarifying pathology associated with movement of the ankle joint during the gait cycle based on changes in the joint space width in feet exhibiting disorders.

Low tibial osteotomy is undertaken for the purpose of maintaining the joint in the early stage of osteoarthritis of the ankle. We believe that low tibial osteotomy results in changes in the alignment of the ankle joint as well as movement of the ankle joint after surgery, similar to the movement noted in healthy feet. Therefore, our method can be used to evaluate the efficacy of the operation and select new operative procedures.

There are several limitations associated with this study. It is necessary to take into account sex and race when evaluating the joint space width. Jonsson *et al.* and Farsø *et al.* reported that the sex-determined difference in the joint space width of the ankle joint is significant [8, 9]. We therefore compared the amount of change in dorsiflexion and plantar flexion relative to that observed in the neutral position. We believe that it is possible to evaluate the changes in the joint space width in detail, however it is also important to investigate the effects of sex and physique on the amount of change in additional cases.

The foot and ankle is a weight-bearing joint, and it is desirable to evaluate the joint space width under loading conditions. In recent years, load devices have become widely used to assess 3D kinematics on CT images [12, 13]. However, while it is easy to place a load in the neutral position, it is very difficult to place a load in maximal dorsiflexion or plantar flexion with high reproducibility. Therefore, we used ankle CT images obtained under non-loaded conditions in this study.

## Conclusion

We evaluated the joint space width of the tibiotalar joint in the neutral position, dorsiflexion and plantar flexion of the ankle joint using 3D reconstruction images of CT. The joint space width increased in the order of dorsiflexion, the neutral position and plantarflexion. Regarding the amount of change in dorsiflexion and plantarflexion relative to that observed in the neutral position, there were no significant differences in the middle-middle position. In contrast, there were highly significant differences in the medial-anterior, medial-middle and medial-posterior positions.
